# Tunnel Deformation Inspection via Global Spatial Axis Extraction from 3D Raw Point Cloud

**DOI:** 10.3390/s20236815

**Published:** 2020-11-28

**Authors:** Cheng Yi, Dening Lu, Qian Xie, Jinxuan Xu, Jun Wang

**Affiliations:** 1 College of Mechanical & Electrical Engineering, Nanjing University of Aeronautics and Astronautics, Nanjing 211100, China; yicheng@nuaa.edu.cn (C.Y.); deninglu@gmail.com (D.L.); qianxie@nuaa.edu.cn (Q.X.); 2 College of Computer Science & Technology, Nanjing University of Aeronautics and Astronautics, Nanjing 211100, China; jinxuanxu027@gmail.com

**Keywords:** raw LiDAR data, central axis extraction, cross section, tunnel deformation, LiDAR sensor

## Abstract

Global inspection of large-scale tunnels is a fundamental yet challenging task to ensure the structural stability of tunnels and driving safety. Advanced LiDAR scanners, which sample tunnels into 3D point clouds, are making their debut in the Tunnel Deformation Inspection (TDI). However, the acquired raw point clouds inevitably possess noticeable occlusions, missing areas, and noise/outliers. Considering the tunnel as a geometrical sweeping feature, we propose an effective tunnel deformation inspection algorithm by extracting the global spatial axis from the poor-quality raw point cloud. Essentially, we convert tunnel axis extraction into an iterative fitting optimization problem. Specifically, given the scanned raw point cloud of a tunnel, the initial design axis is sampled to generate a series of normal planes within the corresponding Frenet frame, followed by intersecting those planes with the tunnel point cloud to yield a sequence of cross sections. By fitting cross sections with circles, the fitted circle centers are approximated with a B-Spline curve, which is considered as an updated axis. The procedure of “circle fitting and B-SPline approximation” repeats iteratively until convergency, that is, the distance of each fitted circle center to the current axis is smaller than a given threshold. By this means, the spatial axis of the tunnel can be accurately obtained. Subsequently, according to the practical mechanism of tunnel deformation, we design a segmentation approach to partition cross sections into meaningful pieces, based on which various inspection parameters can be automatically computed regarding to tunnel deformation. A variety of practical experiments have demonstrated the feasibility and effectiveness of our inspection method.

## 1. Introduction

With the recent advances of 3D scanning sensors, digitalizing large-scale tunnel structures with 3D point clouds is becoming increasingly convenient, which could benefit a variety of applications such as tunnel deformation analysis and 3D modelling. Due to various factors, such as tunnel construction, unexpected earth pressure, and geological conditions, the state of health of the shield structure could be strongly influenced. Furthermore, the most metro tunnel are constructed by shield tunneling machine and the design cross section of the tunnel is circle. The frequent routine inspection of tunnels is a crucial task to determine structural stability [1]. However, accurate deformation analysis to meet the requirements of tunnel gauge and driving safety still remains a challenge in nowadays.

Conventional tunnel inspection techniques often require a considerable amount of time and cannot offer high-density datasets [2,3,4]. 3D laser scanning can provide alternatives to sense the state of a tunnel during its lifetime. At present, the methods of terrestrial scanning and mobile laser scanning measurement are used for data acquisition to monitor tunnel deformation. Terrestrial laser scanning method is widely used for its advantages of high-accuracy data and high location precision in data collection. Multiple stations are required to scan a long tunnel and must be registered. The mobile laser scanning technology is now becoming popular in tunnel monitoring with its rapid, accurate and convenient measurement advantages. Due to the drift of inertial measurement unit (IMU), in underground space, the accuracy of mobile laser scanning system will be affected. Thus, it is mainly used for maintenance and measurement tasks of the tunnel operation period. However, in the post-completion and local key area structures of high precision measurement requirements, terrestrial scanning is still an important construction management and assessment tool. The accessories (e.g., pipelines and cables, bolt holes, and metal stents) are attached very closely to tunnel surfaces. As a result, the raw scanned data are with noticeable noise/outliers and the missing of large regions. Moreover, the actual axis of a tunnel can be hardly the same with its design axis, which is typically the foundation of the cross-sections extraction and further deformation analysis. However, the accurate and automatic extraction of tunnel lining cross-sections directly to reflect the overall deformation behaviors of metro tunnel over raw point cloud data remains a notorious challenge.

Based on the design model, a tunnel can be constructed by sweeping a 2D profile along a spatial path (axis) [5]. The profile section remains perpendicular to the path at all times. Most methods usually fit the tunnel to a typical geometric shape (e.g., circular cylinder or elliptic cylinder) with a least-squares algorithm to extract of tunnel axis before analyzing deformation [6,7,8,9]. The tunnel deformation is estimated by the comparison between the approximation of the vertical section extracted and the theoretical design section at the same position. Many local details may be neglected by these methods during the inspection procedure. As a result, they have limited accuracy in analyzing the global deformation state of large-scale and long tunnel. In summary, the abundant spatial information of the raw LiDAR dataset has not been fully mined, and its capability in deformation analysis inspection has not been maximized.

**Figure 1:**
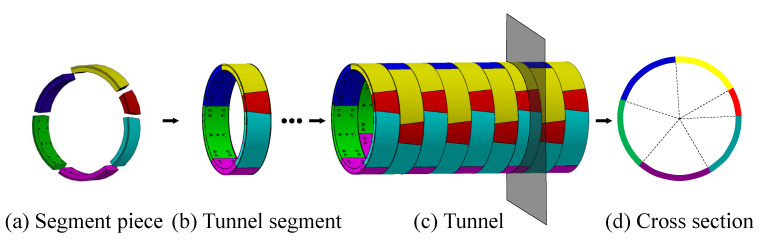
Illustration of the structure of the metro tunnel. The whole tunnel (**c**) is composed of numerous tunnel segments (**b**), which contain several segment pieces (**a**) with fixed arrangement (**d**).

**Figure 2:**
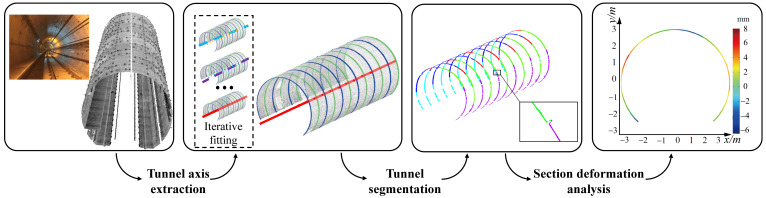
Overview of the proposed tunnel deformation analysis framework from scanned 3D point clouds. It essentially consists of three kernel stages, i.e., tunnel axis extraction, tunnel section segmentation and section deformation analysis.

In this paper, we propose a novel global approach to inspect the tunnel deformation accurately from raw LiDAR point data, by fully exploring the geometric information of the tunnel assembly structures. Our key observation is that the actual axis of a tunnel is a spatial path with which the tunnel profile sweeps along. As illustrated in Figure 1, a tunnel is constructed by tunnel segments, and a tunnel segment contains a certain number (generally six) of segment pieces. Our method comprises of three steps, as shown in Figure 2. Given a tunnel point cloud as input, the design axis is initially exploited to generate a sequence of cross sections on the point cloud. By fitting the cross sections into circles, the centers of fitted circles are approximated with a B-Spline curve, which is considered as an updated axis. The procedure of “circle-fitting and B-SPline approximation” repeats iteratively until convergency, that is, the distance of each fitted center to the current axis is shorter than a given threshold. By this means, the spatial axis of the tunnel can be accurately extracted. With the extracted actual axis, we divide the tunnel ring line of each cross-section into different segments. We can further segment tunnel surfaces by employing the geometric information of assembled pieces, which enables random cross-sectioning and cross-section lines segmentation. The segmented ring lines can be utilized to perform deformation analysis which is achieved by computing displacements in the polar coordinate system.

Extensive experiments manifest the effectiveness of our method, and show that it can produce better deformation analysis than state-of-the-art approaches. Our contributions are summarized as follows.

- We propose an effective method to inspect the deformation of a tunnel from 3D raw LiDAR point clouds. It is capable of analyzing the deformation accurately for large-scale data, as well as in the presence of noticeable noise and outliers. It has been successfully deployed for tunnel inspection on more than ten Metro subway companies.
- We devise an extraction method of the global central axis of the tunnel from 3D LiDAR point data, by mining the original design pattern, which is able to extract the accurate tunnel cross sections in fidelity relative to the original tunnels.
- We design a segmentation algorithm to locate and generate segments of tunnel ring lines, and further to produce tunnel segment pieces.

## 2. Related Work

There exists a number of literature on tunnel monitoring analysis and modeling, ranging from photogrammetric-based methods [10,11,12] to 3D point cloud-based [13,14,15,16,17,18,19,20] ones. We only review previous works most related to our work, including tunnel central axis estimation and cross-section extraction techniques for structure safety evaluation.

Tunnel deformation inspection. The cross-sections of the tunnel, representing the geometric shapes of tunnel linings, can be used to evaluate structural stability. Based on the extracted vertical cross-sections, the tunnel deformation is estimated by comparing vertical sections extracted at the same position from different epochs of point clouds, or contrasting measured vertical sections and theoretical sections. As a result, tunnels can be repaired with the estimated location and degree of deformation during inspection. The photogrammetric methods utilize stereo images taken at various positions, which can obtain the 3D coordinates of the tunnel profile by triangulation [21]. Ukai [22] developed a tunnel deformation monitoring system with line sensor cameras capable of taking high-precision panoramic annular images of tunnel lining surfaces. Wang et al. [23] presented a profile-image method and an improvement method, which overcome the restriction of the number of points of conventional geodetic surveying. However, the precision for the photogrammetric-based approaches are highly dependent on the factors including distortion of lens, number of calibration points, and the visibility conditions in an actual tunnel.

Thanks to TLS, it is possible to obtain quality data with high location precision for the extraction of tunnel cross-sections, even under disadvantageous conditions. Several studies performed tunnel deformation measurement and analysis using the LiDAR data. Lindenbergh et al. [13] introduced a workflow for terrestrial laser scanning to detect deformation and estimate the measurement accuracy. Seo et al. [24] proposed a tunnel cross-section management system to determine arbitrary cross sections of tunnels more promptly and accurately. Researchers adopted a typical geometric model to approximate tunnel datasets, and then estimated cross-sections from the fitted models [25]. However, the accuracy of a tunnel is affected by outliers from tunnel accessories and neglecting local anomaly details, and may be degraded during the modeling process. The approximation of the reconstruction model to the actual tunnel degrades the accuracy of the cross-section estimation and many local anomalies details also may be neglected. Arastounia [26] proposes a fully-automated methods for identifing the tunnel’s main axis and extracting the cross sections, which are acquired by a Velodyne HDL 32E device. It provides a very precise and accurate as-built model of the tunnel for the planning of future design enhancement. Qiu et al. [27] presented an automated method based on laser and machine vision to measure tunnel deformation of multiple interest points in real time and effectively compensate for the environment vibration. Sun et al. [28,29] proposed a scheme for a tunnel monitoring and measuring system with laser scanning as the main sensor for tunnel environmental disease and deformation analysis in the tunnel operation period. Cheng et al. [30] presented an automated and efficient method for extracting tunnel cross-sections. It used a vertical cross-sectional plane to generate tunnel cross-sections based on the central axis of the tunnel. Tunnel cross-sections were generated, by projecting the nearby points onto this plane. In most cases, the tunnel cross-sections from various complicated tunnel scenes cannot be extracted accurately if only geometric information is used. According to the tunnel design standards, the tunnel design axis is composed of plane line and vertical section slope, in which the tunnel horizontal axis should be composed of a series of complex curves of straight line, transition curve and circular curve. Due to the influence of assembly quality during tunnel construction, the tunnel surface cannot be expressed by a unified surface equation. Thus, the proposed method extracts the cross sections from a 3D model of the tunnel, and uses standard geometric models such as a cylinder or a 3D mesh model, to approximate a geometric domain. However, the global geometric fitting method will smooth out the local deformation error. In contrast, our tunnel deformation method extracts tunnel cross-sections from the captured point data, by discovering the regularities of tunnel segment pieces and geometric properties. As a consequence, the abundant spatial information in LiDAR datasets can be fully exploited to monitor tunnel safety, and therefore faithful tunnel deformation analysis is achieved.

Central axis extraction. The key to tunnel deformation analysis is to extract the cross-section points of the tunnel, based on exploring and utilizing the inherent characteristics of tunnel design and construction as prior knowledge, especially by making the cross-section orthogonal to the tunnel. A few methods of fixing a tunnel centerline have been specifically designed, on the basis of the cross sections extracted. However, there often are discrepancies between the design centerline and an actual tunnel central axis. Nuttens et al. [31] proposed an ovalization monitoring methodology to determine the cross-sections of the tunnel, and defines the tunnel axis based on a best-fit cylinder. This method has limited accuracy for curved tunnel deformation. Han et al. [32] projected the point cloud of the tunnel onto a horizontal plane to produce a binary image which was skeletonized to estimate the tunnel centerline. However, it is apt to be overfitted to the saw-like pixels. Kang et al. [33] extracted the continuous central axis of the tunnel using a 2D projection of the point cloud and curve fitting using the RANSAC algorithm. The axis is also optimized using a global extraction strategy based on segment-wise fitting. Puente et al. [34] proposed a tunnel extraction algorithm that was determined by the normal vectors of the tunnel point cloud and the cutting plane was perpendicular to the tunnel axis. Xu et al. [35] developed a sectional projection method to calculate the central axis, which segments the point cloud data of the tunnel structure into thin sections and acquires a projection plane in each section profile. The method is employed to extract and analyze the point clouds of curved tunnels to improve the accuracy. In general, no clear definitions of the tunnel axis are given in these methodologies. By contrast, we propose an exact method of global central axis of the tunnel, by investigating the intrinsic design pattern of tunnels. Our method enables automatic extraction of cross-sections for various complex tunnel scenes.

## 3. Overview

Our method takes the raw LiDAR scanned data of subway tunnels and theoretical axes of tunnel design as input. On top of the design axis, a sequence of cross sections on the point cloud can be extracted. Through fitting the cross sections into circles, the centers of fitted circles are approximated with a B-Spline curve, which is considered as an updated axis. By the procedure repeats iteratively until convergency, the spatial axis of the tunnel can be accurately extracted. Then, a tunnel point cloud segmentation algorithm is proposed to extract the cross section based on the actual tunnel axis. Finally, according to the practical mechanism of tunnel deformation, we design a segmentation approach to partition the tunnel ring lines of cross sections into meaningful pieces, based on which various inspection parameters can be automatically computed regarding to tunnel deformation. Figure 2 shows an overview of the proposed tunnel deformation analysis algorithm, which essentially consists of three steps: (1) tunnel axis extraction, (2) tunnel segmentation, and (3) section deformation analysis.

## 4. Method

### 4.1. Tunnel Axis Extraction

The input of this step is the captured point data $T_{p}$ of a tunnel, and the output is its actual tunnel axis *A*. Intuitively, the geometric model of a tunnel can be reconstructed as a solid volume via the vertical sweeping of a cross-section along the axis. The angle between the cross-section and the sweeping path should be always $90^{\circ }$. To achieve the tunnel axis, we need to first compute each cross-section and further the center of each cross-section. For a point $q_{i}$ on the design axis $A_{0}$ and the corresponding tangent unit vector *T*, we can define a tunnel “cross-section” chunk *P* by Equation (1).

(1) $$P=\{p_{i}\in T_{p}|\;\;|\frac{(p_{i}-q_{i})\cdot T}{∥p_{i}-q_{i}∥}|<\varepsilon \},$$

where ε is the threshold that is set to be 0.05 in default. In fact, Equation (1) searches points within a small angle along the design axis, since points are sparse in the captured LiDAR data and a pure cross-section involves few points.

**Figure 3:**
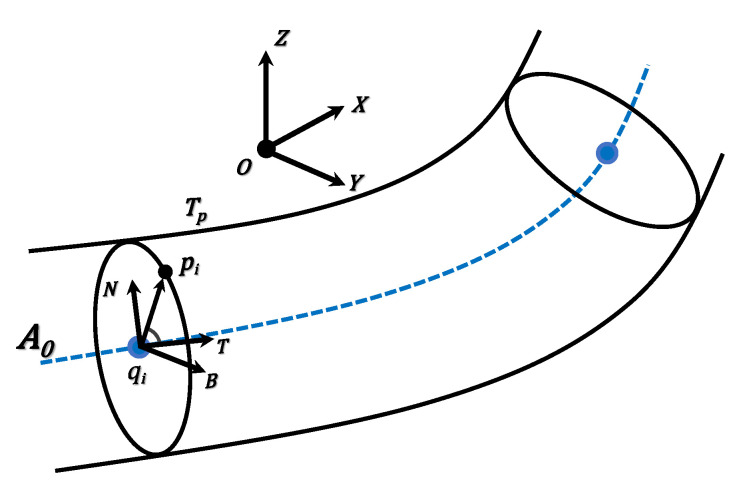
The Frenet frame of the spatial axis of the tunnel.

**Figure 4:**
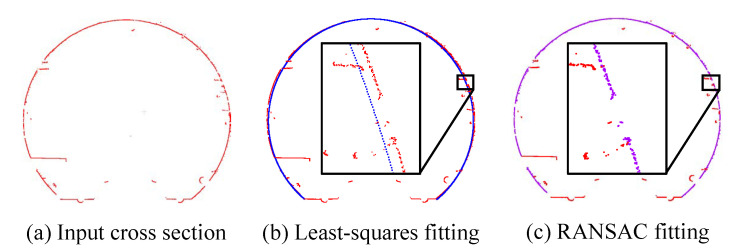
Illustration of different fitting methods on the tunnel section contour point cloud. The input is the tunnel cross section (**a**) extracted from the raw point cloud. Owing to the effect of noisy points inside the tunnel, the RANSAC based method (**c**) achieves better fitting results than Least-square method (**b**).

After obtaining the points of a cross section, we convert them into a local coordinate system – Frenet coordinate system, for convenience and simplicity. Precisely, the normal vector *n* and point *p* define the cross-section plane, as well as the Frenet coordinate system, where T,N,B are the tangent, normal, and binormal unit vectors, as shown in Figure 3. Afterwards, the local coordinates are projected onto the cross-section plane to generate a 2D section which is noisy due to the presence of cables, pipelines, etc. To alleviate these issues, we employ the RANSAC based circle fitting algorithm [36] to remove such noise/outliers from the achieved 2D section. Compared with least-squares fitting, the RANSAC algorithm has better robustness in tunnel point cloud denoising, as shown in Figure 4. We still denote the resulting 2D points by *P*.

We obtain the optimal center *c* of a 2D section by minimizing the sum of squared distances from the center *c* to points on the section.

(2) $$c^{*}=\arg\min_{c}\sum_{p_{j}\in P}(∥p_{j}-c{∥-r)}^{2},$$

where $c^{*}$ is the optimized center of the given section. The radius *r* denotes the distance from the design tunnel axis to the tunnel surface.

Equation (2) can be effectively solved via the gradient descent algorithm. Based on the 3D point cloud data, the actual tunnel axis can be constructed with a series of cross-section centers, which can be formulated as:

(3) $$A=G(c_{1},c_{2},c_{3},\cdots ,c_{n}),$$

where G(·) is a construction function for the tunnel axis, and $c_{i}$ is the center of the *i*-th tunnel section.

It is impractical to straightforwardly connect those centers by line segments, since a tunnel usually has no sudden tunnel changes along the axis. Thus, it is necessary to enable the smoothness of the axis. B-spline, which is locally modifiable, is an intuitive choice, and we introduce a B-spline tunnel axis fitting scheme. We first obtain a preliminary fitting based on algebraic distance, which is formulated as

(4) $$minI\left(D\right)=\min\sum_{i=1}^{n}{\left(\sum_{j=0}^{k}N_{j,3}\left(u_{i}\right)\cdot d_{j}-c_{i}\right)}^{2},$$

where $N_{j,3}\left(u_{i}\right)$ is a cubic B-spline basis function. $u_{i}$ is the node value of a section center $c_{i}$ parameterized by the cumulative chord length and $D={\left\{\begin{array}{c} d_{j} \end{array}\right\}}_{j=0}^{k}$ are the control points.

The above formula is a linear least-squares problem, which can be solved. In order to make the curve fitted by B-spline more closer to the actual axis, we introduce the method of Least-squares orthogonal distance fitting to update each section center.

(5) $$\begin{array}{c} minII\left(D\right)=min\sum_{i=1}^{n}\left|\right|\left(\begin{array}{c} x_{i}\\ y_{i}\\ z_{i} \end{array}\right)-\sum_{j=0}^{k}N_{j,3}\left(u_{i}\right)\cdot \left(\begin{array}{c} d_{jx}\\ d_{jy}\\ d_{jz} \end{array}\right){\left|\right|}^{2} \end{array},$$

where $D={\left(u_{1},u_{2},\cdots ,u_{n},d_{0x},d_{0y},d_{0z},\cdots ,d_{kx},d_{ky},d_{kz}\right)}^{T}$ is the node point and control point vector. $c_{i}={\left(x_{i},y_{i},z_{i}\right)}^{T}$ is the center point. The above equation can be solved by the Gauss-Newton method. The initial value of $u_{i}$ iteration is the node value parameterized by the cumulative chord length, and the initial values of $d_{jx},d_{jy},d_{jz}$ are the fitting results based on the above algebraic distance. By this meams, a smooth center axis can be extracted.

**Figure 5:**
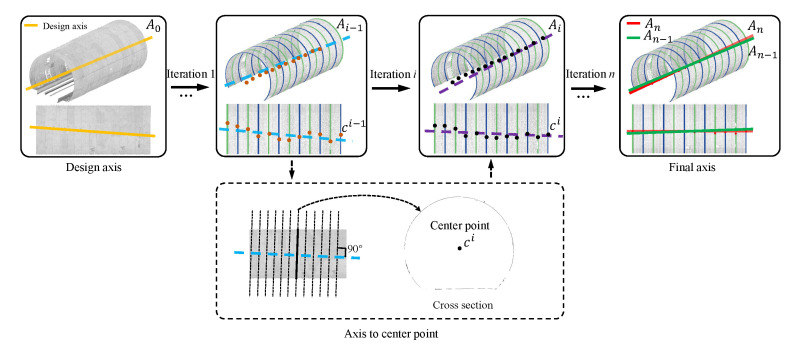
Illustration on iterations of tunnel axis extraction. The designed axis is used as the initialization state of the iteration. At the beginning of the iteration, the center points are first generated according to the designed axis. With the generated center points, a new fitted axis can then be obtained, which replaces the designed axis. We then repeat the above operation until we get a stable fitted axis between two iterations. That is, the coverage of the iteration is that the error between two fitted axes is below a threshold.

The error of two consecutive iterations of the actual axis can be calculated as

(6) $$e=\sum_{i=1}^{n}{∥s_{i}-s_{i}^{\prime }∥}^{2},$$

where $s_{i}$ is the *i*-th sampling point of $A_{i}$. We construct the section plane $pln_{i}$ on $s_{i}$, which is perpendicular to the $A_{i}$. We intersect $pln_{i}$ to $A_{i-1}$, to obtain the intersection point $s_{i}^{\prime }$. μ is a pre-defined threshold (e.g., 0.03 m). If e>μ, we repeat the above process and calculate the error until e≤μ (i.e., converged), $c^{i}$ is the corresponding center of a 2D cross section, as illustrated in Figure 5. The procedure is shown in Algorithm 1.
**Algorithm 1** Tunnel Axis Extraction.**Require:** Raw point cloud $T_{P}=\left\{p_{i}\right\},i=\left\{1,\cdots ,N\right\},$ Tunnel design axis $A_{0}$. **Ensure:** Tunnel actual axis $A_{n}$
1:**function**ITERARIVE TUNNEL AXIS FITTING($T_{P},A_{i}$)  2:    Initialization:  3:    i=0.  4:    **while** (e<μ) **do**  5:        $A_{n}=A_{i}$;  6:        i=i+1;  7:        *P*←Get Section Set $\left(T_{P},A_{i-1}\right)$;  8:        *C*←Get Section Center Set(P);  9:        $A_{i}$=Algebraic Distance based Curve Fitting I(C);  10:        $A_{i}$=Orthogonal Distance based Curve Fitting $II(A_{i},C)$;  11:        e=Error Computing $\left(A_{i-1},A_{i}\right)$;  12:    **end while**  **return** $\left(A_{n}\right)$;  13:**end function** 


### 4.2. Tunnel Line and Surface Segmentation

A subway tunnel is constructed by multiple segments along the tunnel axis (Figure 1). Given the actual tunnel axis output by Section 4.1, multiple tunnel segment rings can be defined along the axis, by setting a uniform distance ϵ which is the typical tunnel segment width (1.5 m). Then the point cloud ψ of each tunnel segment ring can be extracted from the original point cloud data. Characteristics like sags can usually provide evidence for segmentation along each ring. Nonetheless, it is not accurate since there always exist noise/outliers due to accessories like pipelines and cables.

With the obtained point cloud of a tunnel segment ring, we first compute the projected points of the cross section according to Section 4.1. We then exclude the sparse points that have less than 5 neighbors.

We further apply PCA [37] to calculate normals for the projected 2D points. We define the angle between a vector from a point of these 2D project points to the center and the normal of this point as

(7) $$a_{i}=acos\left(n_{i}\cdot \left(p_{i}-c\right)\right),$$

where $n_{i}$ is the normal vector of the projected point $p_{i}$, and *c* is the center calculated acoording to Section 4.1.

**Figure 6:**
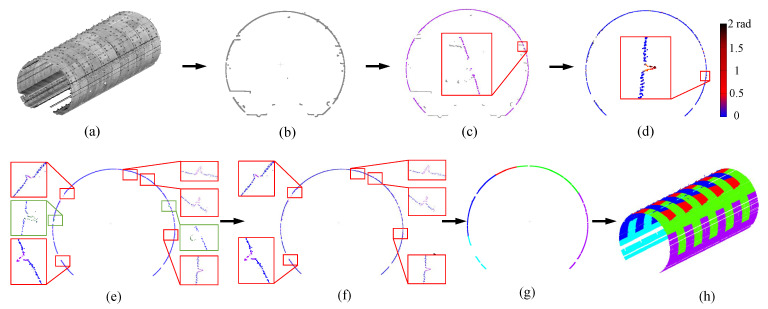
Tunnel line and surface segmentation. (**a**) Input raw LiDAR scan; (**b**) Tunnel section interception; (**c**) RANSAC denoising; (**d**) Normal vector and center angle quantification; (**e**) Extracting different geometric feature; (**f**) Retaining pipe seamdetetor feature; (**g**) Section segmentation result; (**h**) Segmentation result returns to the point cloud space.

Note that the angle at the segment connection position is typically larger than other locations. However, we also found that the accessories could bring ambiguity as they could also produce great angles. As shown in Figure 6e, the accessories points are colored in green, the segment connection points are colored in red. To alleviate this issue, we first use k-means to cluster the candidate points for possible connection locations obtained by Equation (7), and obtain a group within a distance, defined as follows:

(8) dist=50l,

where *l* is the minimum distance of the tunnel point cloud(e.g., 0.1 mm in our experiments). For each point set $cls_{i}$, we calculate its center $g_{i}$ by averaging the involved points, and perform a sphere neighborhood search (r=5dist) and compute $g_{i}{}^{\prime }$ by averaging the neighbors.

The distinction between connections and accessories can be defined as

(9) $$\omega_{i}=\left\{\begin{array}{cc} 1, & \mathrm{if}\;\left(g_{i}-g_{i}{}^{\prime }\right)\cdot \left(g_{i}-o\right)>0\\ 0, & else \end{array}\right.$$

where $\omega_{i}$ is the computed indicator of each point in $P{}^{\prime }$. This is because pipe seamdetetors are outer while the accessories are inner, from a comparison perspective. By this means, we can remove the accessories points, as shown in Figure 6f.

Besides the line segmentation, we also do the surface segmentation to enable random cross-sectioning on the tunnel. In essence, it is to extract the boundary lines between two adjacent segments of a tunnel segment ring. To do so, we extend [38] to address this issue. In particular, we first generate proposals (i.e., straight line) for the 3D boundaries, and then judge the proposals with a quality function.

Considering our context, we present the quality function below.

(10) $$Q\left(h,\Phi ,\epsilon \right)=\left|\Phi \right|-\sum_{\phi \in \Phi }min\left(1,max\left(\frac{\varphi {\left(h,\phi \right)}^{2}}{\Upsilon {\left(\epsilon \right)}^{2}},1-\frac{\varphi {\left(h_{\Delta },\phi \right)}^{2}}{\Upsilon {\left(\epsilon \right)}^{2}}\right)\right),$$

where |Φ| is the total number of 3D boundary points by the geometric feature points of cross section, and ϕ is any point in the set Φ. φ(.,.) is a distance function that computes the Euclidean distance from a 3D boundary point to a proposal *h*. $h_{\Delta }$ is the the compound model instance. ϵ is the user-defined threshold, Υ(ϵ) is the tolerance function in order to take more points into account during sampling step, and we here set Υ(ϵ)=1.5ϵ.

We adopt a confidence-based condition for termination, by ensuring

(11) $$1-\eta \leq (1-{{\left(\frac{\left|\theta \right|}{\left|\Phi \right|}\right)}^{m})}^{\kappa },$$

where η is the pre-defined confidence(e.g., 0.99), and |θ| is the number of inliers. *m* is the minimum number with its default value being 2, and κ is the number of iterations. With setting κ, we can successfully extract boundary lines, among which the *i*-th boundary line can be denoted by $L_{i}={\left\{\phi_{ij}\right\}}_{j=1}^{m}$, as shown in Figure 6.

### 4.3. Deformation Analysis

**Figure 7:**
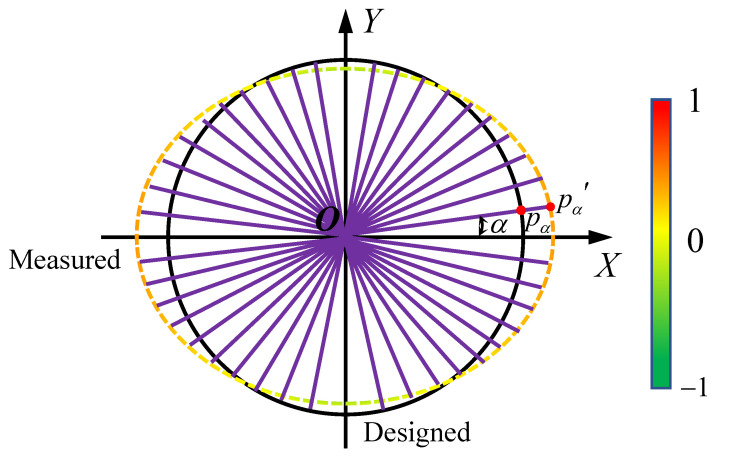
Illustration of the deformation analysis of the scanned cross section, compared with the designed circle pattern. The distance from the scanned to the designed is scaled to [−1,1], and colorized according to the color bar. Negative distance means the scanned point is inside the designed circle.

At the final step, we conduct the deformation analysis based on the above two steps. As mentioned above, surface segmentation enables random cross-sectioning on the tunnel for users. As illustrated in Figure 7, we design a metric to evaluate the displacement μ between the actual tunnel line segments and its designed tunnel ring via cross sectioning.

(12) $$\;\mu =\frac{p_{\alpha }-O}{\left|\right|p_{\alpha }-O\left|\right|}\cdot \left(p_{\alpha }{}^{\prime }-p_{\alpha }\right),$$

where $p_{\alpha }$ is the point with an angle of α in the polar coordinate system of the projected plane, and *O* is the center after aligning the designed section to the actual section, and $p_{\alpha }{}^{\prime }$ is the point of the angle α in the designed tunnel ring.

Similarly, we also need to do alignment for the same actual tunnel line at different times, for further deformation analysis. Besides, the deformation analysis for two neighboring tunnel lines produced at the same time also can be analyzed. To realize this, we obtain the two cross sections located in the both sides of the segment seam with equal distance. Then we project them to 2D planes alone the tunnel axis respectively, followed by aligning them based on the section centers.

## 5. Results

**Figure 8:**
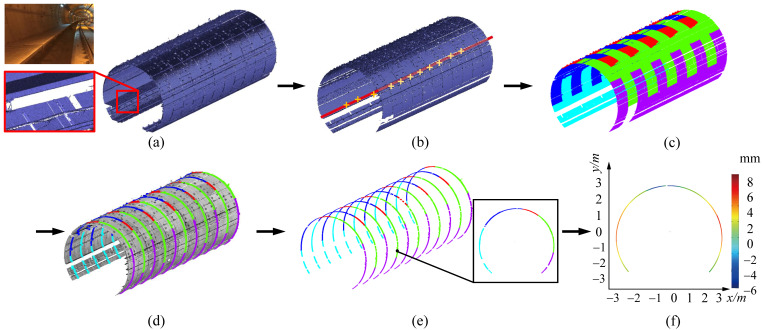
Tunnel point cloud segmentation and deformation analysis in case of significant occlusions. (**a**) The raw scan with large missing regions; (**b**) The tunnel axis extraction; (**c**) The segmentation result of the raw data; (**d**,**e**) A series of cross sections captured through the extracted axis; (**f**) The quantitative deformation analysis of the cross-section. From the results, even with significant occlusions, our method is able to achieve accurate segmentation and analysis.

**Figure 9:**
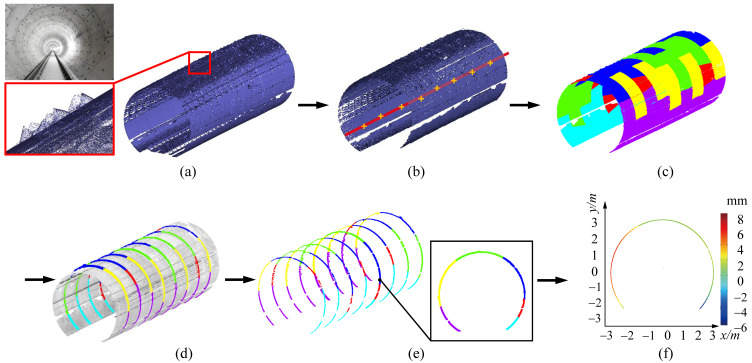
Tunnel point cloud segmentation and deformation analysis in case of severe noise and outliers. (**a**) The raw scan with noise and outliers; (**b**) The tunnel axis extraction; (**c**) The segmentation result of the raw data; (**d**,**e**) A series of cross-sections captured through the extracted axis; (**f**) The quantitative deformation analysis of the cross-section. Note that our method can cope with the raw data with high level of noise and outliers.

**Figure 10:**
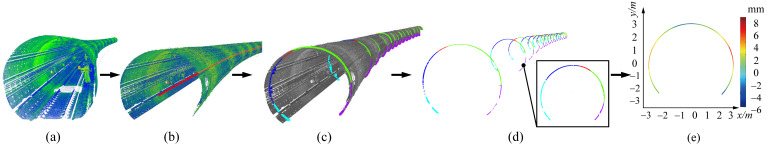
Large scale Tunnel point cloud segmentation and deformation analysis. (**a**) The large raw LiDAR scan with more than 5 km and 1 billion points; (**b**) The tunnel axis extraction; (**c**,**d**) A series of cross-sections captured through the extracted axis; (**e**) The quantitative deformation analysis of the cross-section. Our method achieves more than 3000 tunnel segment segmentation accurately, and further deformation analysis.

In this section, we present a variety of visual results and quantitative results, to validate our method and show the superiority over other methods. The experiment was conducted in the tunnel of Shenzhen (Guangdong, China) Metro Line 1 and Line 3 in to verify the deformation analysis. The tunnel is a circular shield tunnel in the operation stage. The design width of the circular segment in the experimental area is 1.5 m. The Metro Line 1 and Line 3 have operated safely for eight years. We conducted the first 3D laser scanning of the tunnel structure in September 2018. To analyse the deformation of the tunnel, we carried out a second 3D laser scanning of the same tunnel structure in September 2019. The raw LiDAR point scans in our experiments are all scanned by a FARO Focus3D 330 laser scanner with high resolution. The registration of multiple stations data is realized by setting cross-shaped targets. The tunnel segmentation and deformation analysis method can be evaluated through a number of raw LiDAR point clouds, which are always characterized by various noise and occlusions. First, we focus on the accuracy of our segmentation algorithm, especially in some defective cases, where accuracy can be measured by several metrics in Section 5.3. Second, to test the stability of our system, we apply the deformation analysis method to a series of tunnel sections with various complexities and styles, as demonstrated in Figure 8, Figure 9 and Figure 10. As can be seen, our method manifests pleasing performance in all steps without intervention, resulting in promising accurate deformation analysis of the tunnel.

### 5.1. Tunnel LiDAR Data

Occlusions. In Figure 8, we test our method on the raw LiDAR data with missing regions, due to occlusions from the accessories like pipelines and cable. Since our segmentation algorithm using the pipe connection area, it is hardly affected by the occlusions. As a result, our method successfully splits the each tunnel ring line into segments, and then the surface segmentation can be achieved. Furthermore, for a cross-section of the tunnel, our method is able to fill up the missing places of each segment piece, which enables the accurate deformation analysis. It has been shown in Figure 8c,d. Thus, even with severe missing regions, our method is still able to achieve the accurate tunnel segmentation as demonstrated. In Figure 8e, we analyze the deformation of the tunnel cross-section against the design model visually and quantitatively. Due to the unbalanced force, the tunnel tends to deform irregularly. As a result, the deformation varies in different orientations in the cross-section. The greatest deformation lies at the top and both sides of the section, which is consistent with the pattern of material mechanics.

Noise and outliers. We also evaluate our approach on the raw LiDAR scan corrupted with noticeable noise and outliers. As can be seen in Figure 9, our method is fairly robust to noise and outliers, and segmentation can be accurately achieved. In Figure 9e, the deformation analysis is applied to the segmented lines, and we can see that the tunnel section tends to be roughly elliptical according to the deformation statistics. Especially, the deformation of the tunnel mostly occurs in the riveting position of the assembly between the segment pieces. As can be seen, the tunnel deformation is irregular.

Large-scale data. Figure 10 demonstrates that our method is capable of dealing with large-scale, raw LiDAR point data (more than one billion points). The input data in Figure 10a is captured from a tunnel with five kilometers, which involves straight and curved parts. The curved part data usually involves missing regions. However, our approach can still extract a global axis of the tunnel (Section 4.1), which ensures accurate cross-sections in the curved part. Figure 10 manifests excellent performance in tunnel segmentation and deformation analysis, in terms of large-scale point data.

### 5.2. Comparisons

Different from the most related methods that employ circle fitting or ellipse fitting to the tunnel cross-section directly, our approach has more geometrical fidelity to the underlying characteristics of tunnel deformation. For fair comparisons, the corresponding parameters of circle and ellipse fitting are fine tuned to achieve the best results. We adopt two metrics–distance error, RMSE (Root Mean Square Error) and PGP (Proportion of Good Points), which are defined in Section 5.3.

**Figure 11:**
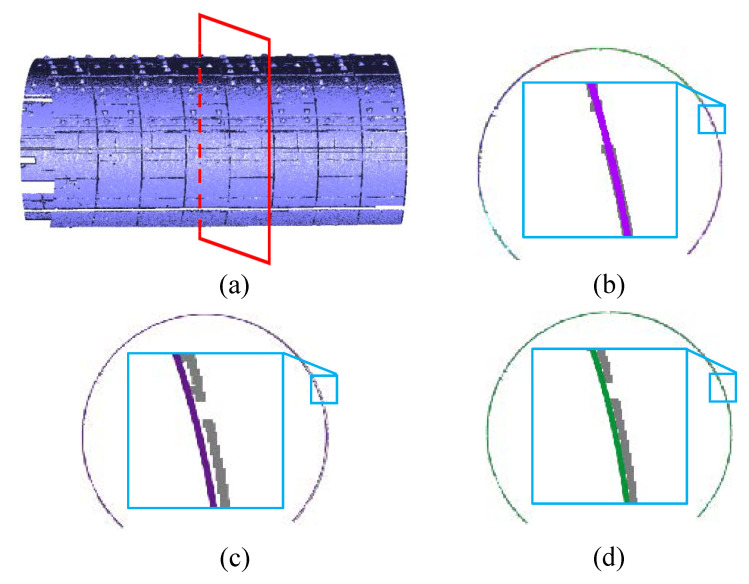
We can extract a cross section(**a**) at any location from the raw point cloud. Comparison on cross-section fitting method of (**b**) our method to (**c**) circle fitting and (**d**) ellipse fitting. From the result, our section fitting strategy obtains visibly best performance.

**Figure 12:**
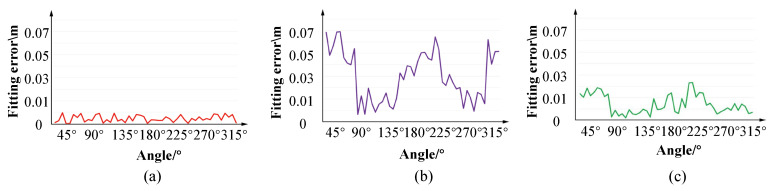
Quantitative comparison of section fitting from (**a**) ours, (**b**) circle fitting and (**c**) ellipse fitting, where the horizontal axis stands for the different angles of the section, and the vertical axis is fitting error calculated in Section 4.3. We can see that our method achieves more accurate result than circle and ellipse fitting.

As mentioned before, because of unbalanced force, the tunnel cross-sections often suffer from unpredictable deformation rather than simply changing into an ellipse. As a consequence, circle fitting and ellipse fitting methods tend to result in large errors, which would further pose negative impacts to deformation analysis. According to the design of tunnels, we learn that deformation usually occurs at the connections of the adjacent segments. Figure 11 and Figure 12 demonstrate that the error of circle fitting is larger than that of ellipse fitting. Meanwhile, our method obtains the best result among these three methods, in terms of quality and quantity.

**Figure 13:**
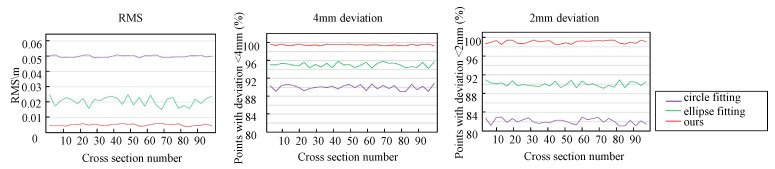
Quantitative section fitting comparison through RMSE (Root Mean Square Error) and PGP (Proportion of Good Points). From the results, our method outperforms circle and ellipse fitting, in terms of three quantitative metrics.

In addition, we also compare our approach with a more recent piecewise fitting method [20] which utilizes the image segmentation to achieve the surface segmentation, as shown in Table 1 and Figure 13. As the image segmentation method is based on fixed template matching, it is impractical to segment the surface accurately in the presence of relatively large displacements. Since our method utilizes the faithful geometric information, it outperforms that method.

### 5.3. Quantitative Evaluation

To measure the accuracy of our method, we use several common quantitative metrics. As illustrated in Figure 7, the error of a fitting result against the measured data is defined.

Besides the fitting error, we also utilize the RMSE (Root Mean Square Error) and PGP (Proportion of Good Points) to measure the geometric fidelity of the fitting results. RMSE and PGP can be defined as:

(13) $$\mathrm{RMSE}=\sqrt{\frac{\sum_{\alpha =1}^{m}{\left(∥p_{\alpha }-{p_{\alpha }}^{\prime }∥\right)}^{2}}{m}},$$

(14) $$PGP_{d}=\frac{∥I∥}{N},$$

where *N* is the number of the cross-sections N of the raw data, and $∥\cdot ∥$ means the cardinality of a set, and I is defined by:

(15) $$I=\left\{p\in N|∥p-p^{\prime }∥<d\right\}$$

The quantitative results of our method and other three methods (circle fitting, elliplse fitting and [20]) are summarized in Table 1. As can be seen in Table 1, our method achieves the lowest error among all four approaches. This is because our method can extract the segment pieces precisely based on the faithful geometric information rather than assuming other prior knowledge.

### 5.4. Applications

Based on the proposed tunnel cross section deformation analysis framework, we develop several practical applications in the structural health monitoring of metro tunnel, such as the adjacent sections step measurement and section change inspection with different periods. These applications achieve favorable results, demonstrating the effectiveness and practicability of the proposed framework in the metro structural health monitoring field.

**Figure 14:**
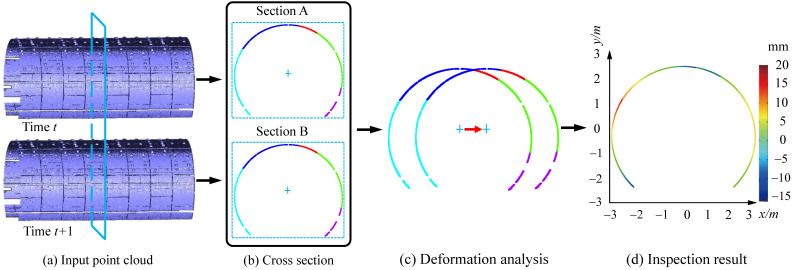
Application in section change inspection with different periods.The first scanning time t is September 2018. A year later t + 1, we performed a second scanning. The tunnel deformation analysis can be achieved by comparing the cross sections of different time.

Section change inspection is a vital aspect reflecting the health status of the tunnel. We first get the scanned point clouds of the same section with different time. The proposed section extraction method is then utilized to obtain the cross sections. The final deformation analysis can be achieved by comparing the cross sections of different time. From the comparison result, we can see that the whole deformation status from time *t* to time t+1, as shown in Figure 14. Generally, the security risks may occur in the tunnel with deformations of more than 10mm. Thus, with the deformation analysis result generated by our method, one can easily distinguish if the deformation is over the maximal deformation threshold. Meanwhile, we may also obtain some hints of the underground force to the tunnel according to the trend of the deformation.

**Figure 15:**
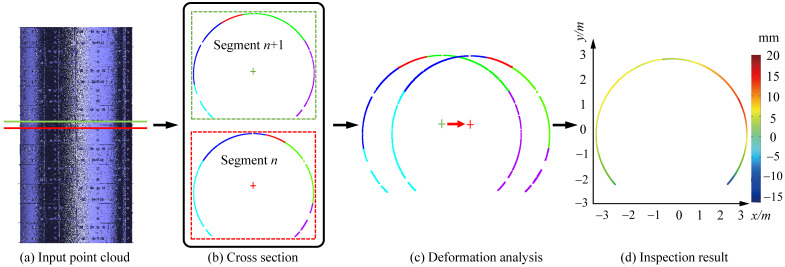
Application in the adjacent sections step measurement.

Figure 15 shows the section step measurement result of two adjacent cross sections. From the figure, we can see the difference between two adjacent cross sections. This comparison result can also offer some information on the health status of the tunnel, since the difference between two adjacent sections should be insignificant if the tunnel is in well condition. From the results, our method manifests favorable performance in tunnel structural health monitoring, which can lead to a relatively high credibility of the deformation analysis.

## 6. Conclusions

For raw LiDAR point clouds of the tunnel, a novel approach for the tunnel deformation analysis have been proposed. We convert tunnel axis extraction into an iterative fitting optimization problem. Given a tunnel point cloud as input, the design axis is initially exploited to generate a sequence of cross sections on the point cloud. By fitting cross sections with circles, the fitted circle centers are approximated with a B-Spline curve, which is considered as an updated axis. The procedure of “circle fitting and B-SPline approximation” repeats iteratively until convergency, that is, the distance of each fitted circle center to the current axis is shorter than a given threshold. By this means, the spatial axis of the tunnel can be accurately achieved. Subsequently, according to the practical mechanism of tunnel deformation, we design a segmentation approach to partition cross sections into meaningful pieces, based on which various inspection parameters can be automatically computed regarding to tunnel deformation. We have demonstrated the feasibility and effectiveness of our inspection method by experimenting on a variety of practical projects, which are used to evaluate the safety state of tunnels. The main limitation of our approach is the point sparsity issue caused by various factors like instrument, light and so on. This would lead to inaccurate segmentation and further deformation analysis, since the key geometric information that we need may be limited or missing. In the future, we would like to exploit and integrate the upsampling into our framework.

## Figures and Tables

**Table 1:** Quantitative benchmark shows the fitting accuracy comparisons on the experimental data sets from circle fitting, ellipse fitting, C. Yi [20] and our method.

Data Sets	Methods	Quantitative Metrics		
		RMSE	${\mathrm{PGP}}_{4\mathrm{mm}}$	${\mathrm{PGP}}_{2\mathrm{mm}}$
Figure 8	Circle fitting	0.048	77%	70%
	Ellipse fitting	0.026	83%	79%
	C. Yi [20]	0.007	94%	90%
	Ours	**0.006**	**97%**	**93%**
Figure 9	Circle fitting	0.051	75%	69%
	Ellipse fitting	0.023	86%	81%
	C. Yi [20]	**0.008**	92%	89%
	Ours	0.008	**95%**	**93%**
Figure 10	Circle fitting	0.054	74%	69%
	Ellipse fitting	0.022	87%	81%
	C. Yi [20]	0.012	90%	85%
	Ours	**0.007**	**95%**	**92%**
